# Downregulation of *TMPRSS4* Enhances Triple-Negative Breast Cancer Cell Radiosensitivity Through Cell Cycle and Cell Apoptosis Process Impairment

**DOI:** 10.31557/APJCP.2019.20.12.3679

**Published:** 2019

**Authors:** Ganiou Assani, Julien Segbo, Xiaoyan Yu, Akadiri Yessoufou, Yudi Xiong, Xiong Zhou, Yunfeng Zhou

**Affiliations:** 1 *Hubei Cancer Clinical Study Center, Hubei Key Laboratory of Tumor Biological Behaviors, *; 2 *Department of Radiation and Medical Oncology, Zhongnan Hospital, Wuhan University, Wuhan, Hubei, China, *; 3 *University of Abomey Calavi, BP 526, Cotonou, Benin. *

**Keywords:** TMPRSS4, Triple-negative breast cancer, Radiosensitivity, Cell cycle, Cell apoptosis

## Abstract

**Background:**

Radioresistance remains a challenge for cancer radiotherapy. The present study aims to investigate the role of *TMPRSS4* in triple negative breast cancer (TNBC) cell radiosensitivity.

**Materials and Methods:**

After transfection of MDA-MD-468 triple negative breast cancer cells line by using the lentivirus vector, the effect of *TMPRSS4 *down-regulation on TNBC radiosensitivity was evaluated by using cloning assay and CCK-8 assay. The CCK-8 assay was also used for performing cell proliferation analysis. Western blot was carried out to detect the expression of certain proteins related to cell cycle pathways (cyclin D1), cell apoptosis pathways (*Bax*, *Bcl2*, and *Caspase3*), DNA damage and DNA damage repair (TRF2, Ku80 , ˠH2AX) . The cell cycle and cell apoptosis were also investigated using flow cytometer analysis.

**Results:**

*TMPRSS4* expression was down-regulated in MDA-MB-468 cells which enhanced MDA-MB-468 cells radiosensitivity.* TMPRSS4* silencing also improved IR induced cell proliferation ability reduction and promoted cell arrested at G2/M phase mediated by 6 Gy IR associated with cyclin D1 expression inhibition. Moreover,* TMPRSS4* inhibition enhanced TNBC apoptosis induced by 6 Gy IR following by over-expression of (Bax, Caspase3) and down-regulation of Bcl2 as the pro-apoptotic and anti-apoptotic proteins, respectively. Otherwise,* TMPRSS4* down-regulation increases DNA damage induced by 6 Gy IR and delays DNA damage repair respectively illustrated by downregulation of TRF2 and permanent increase of Ku80 and ˠH2AX expression at 1 h and 10 h post-IR.

**Conclusion:**

Down-regulation of* TMPRSS4* increases triple negative breast cancer cell radiosensitivity and the use of* TMPRSS4* inhibitor can be encouraged for improving radiotherapy effectiveness in TNBC radioresistant patients.

## Introduction

Breast cancer is cancer frequently diagnosed in about 84% of countries in the world and the leading cause of cancer death in over 54% of world countries (Bray et al., 2018). In 2012, the number of the new cases occur was estimated at 1, 7 million and 520.000 deaths (Torre et al., 2012 and 2016; Lee et al., 2018,Chen et al., 2015) against 2, 1 million and 626.679 death in 2018 (Bray et al., 2018). Based on these data, breast cancer can be considered as an important public health issue and more research have to be encouraged for its preventive and curative treatment. Moreover, Triple negative breast cancer (TNBC), characterized by the lacks of expression of estrogen receptor (ER), progesterone receptor (PR) and human epidermal growth factor receptor2 (HER2) (Lukoseviciene et al., 2018), is the most aggressive among the subtype of breast cancer and represents about 15% of all case of breast cancer (Millikan et al., 2008; Yang et al., 2019; Abramson et al., 2015; Wen et al., 2019). However, due to its molecular characteristics, TNBC remains more difficult to treat with common breast cancer therapies and easily develop resistance to therapy including the radiotherapy (Foulkes et al., 2010; Agrawal et al., 2014; Lehmann et al., 2011; Augoff et al., 2012; Tian et al., 2017; Ilgun et al., 2016) which is useful because it contributes to more than 50% of cancer treatment (Wen et al., 2019; Kindts et al., 2017; Moran et al., 2015). For resolving the radiotherapy resistance problems of TNBC, it is therefore important to explore the new biomarkers that predict or improve the radiotherapy effectiveness.

A single-pass typeII membrane protein transmembrane protease serine4 (TMPRSS4) is a novel serine protease discovered at the cell surface.* TMPRSS4* is highly expressed in several types of cancer cell lines (Chikaishi et al., 2016; Wu et al., 2014; Liang et al., 2013) especially the TNBC cell line (Cheng et al., 2013; Li et al., 2017).* TMPRSS4* is also implicated in cancer cell proliferation (Huang et al., 2014; Kim et al., 2010) and cancer radiotherapy (Li et al., 2011). It has been shown that* TMPRSS4* inhibition reduced cancer cell proliferation ability by promoting cell cycle arrested, cell apoptosis and enhanced radiotherapy effectiveness (Huang et al., 2011; Li et al., 2011; Fan et al., 2018; Lee et al., 2016; Jung et al., 2008). Moreover, cell cycle and cell apoptosis are two steps of cancer progression and are implicated in cancer treatment (Diaz-Moralli et al., 2013; Pistritto et al., 2016; Wong et al., 2011; Kadam et al., 2016) including radiotherapy (Assani et al., 2019; Pawlik et al., 2004; Meyn et al., 1997). However, the function of* TMPRSS4* in cancer cell radiosensitivity especially the triple negative breast cancer remains unclear. In the present study, the stable* TMPRSS4* down-regulating TNBC cell lines were used to explore the role of* TMPRSS4* on triple negative breast cancer radiosensitivity in relationship with cell cycle and cell apoptosis. From our results, we demonstrated the negative correlation between* TMPRSS4* expression and TNBC sensitivity to irradiation suggested that* TMPRSS4* could be considered as a potential therapeutic target for enhancing breast cancer radiotherapy efficacy especially the triple negative breast cancer.

## Materials and Methods

*Cell line and cell culture*

Human breast cancer cell (MDA-MB-468), one of the commonly used for TNBC studies, was obtained from the key laboratory of tumor biological behavior of Hubei province and was cultured in Dulbecco’s modified eagle medium (DMEM, Hyclone, Logan, USA) supplemented with 10% fetal bovine serum (FBS, WiSant,Canada), 100 U/ml penicillin and 100µg/mL streptomycin under 37^o^C with 5% CO_2_ humidified atmosphere. 

*Vector and stably transfected cell lines development*

The lentivirus vectors PLKO.1-sh-TMPRSS4 versus PLKO.1-sh-NC (Bioeagle/Wuhan/China) were transfected in MDA-MB-468 cell lines. The stably transfected cell lines were selected at 5µg/mL puromycin after two to three weeks post-transfection. The stable transfected cells lines developed were respectively named MDA-MB-468-shTMPRSS4 and MDA-MB-468-NC. The expression of* TMPRSS4* was detected at mRNA and protein level by qPCR and western blot, respectively.

*Western blot analysis*

Western blot was implemented for the analysis of protein expression. The stable transfected MDA-MB-468 were washed twice with ice-cold phosphate-buffered saline (PBS) and were treated with radioimmuno-precipitation assay lysis buffer (#P0013B, Beyotime biotechnology, China) containing 1XPMSF (#ST506, Beyotime biotechnology, China) and phosphatase inhibitor tablets (#4906837001, Roche, USA). For western blot assay, the same quantity of extracted protein was electrophoresed with 8 – 12,5 % sodium dodecyl sulfate-polyacrylamide gel (SDS Page) and transfected to polyvinylidene difluoride (PVDF) membrane with the transfer buffer following by the membrane blocking with 5% non-fat milk at room temperature for 2 h. The membranes were incubated with the primary antibodies at 40C overnight and washed three times with Tris-buffered saline containing 0,1% of Tween-20 before its incubation with goat anti-rabbit or anti-mouse horseradish peroxidase-conjugated antibodies (1:10,000; respectively Lot 1387291024 and Lot 1357561012, Cell Signaling Technology) for 1 h at room temperature at least. The Tris-buffered saline containing 0,1% of Tween-20 was used for washing the membranes three to five time( 10 min per washing) prior to each step. The primary antibodies used included: GAPDH (1:5,000,#60004-1-Ig,Proteintech,China),* TMPRSS4* (1:1,000 dilution,#A4865, ABclonal,USA ), Cyclin D1 (1:1,000 dilution,#60186-1-lg, Proteintech,China), *Bax *(1:1,000 dilution, #50599-2-Ig, Proteintech,China ),* Bcl2* (1:1,000 dilution,#A16776, ABclonal,USA), Caspase3 ( 1:1,000 dilution, # 19677-1-AP, Proteintech, China), *ˠH2AX* (1:1,000 dilution,#3222535-1, Abcam) , *Ku80 *(1:1,000 dilution, #A5862#, ABclonal,USA),*TRF2 *(1:1,000 dilution, # 3224856-2, Abcam,USA). For the experiments under irradiation, the proteins were extracted from the irradiated cells with an adequate dose of irradiation and incubated to confluence. The Enhanced Chemiluminescence system ECL ( Adanster, USA) was used to visualize the separated proteins bands and chemical dose XRS + System ( Bio-Rad, Hercules,CA,USA) and Image JProgram were respectively used to capture and analyzed (quantify) the generated images.

*Colony Formation Assay*

The colony formation assay was performed to evaluate the survival fraction of cells with different treatments. The stably transfected cells (300 cells/ 2mL/well) were plated in 6 wells plate. After 24 h post-culture, the cells were irradiated with a single dose of 0, 2 and 4 Gy using the small animal radiation research platform (SARRP, Px I X-RAD 225 Cx, CT, USA, from a 204 Kv photon beam. At 14 days post-incubation, the developed colonies were washed with PBS, fixed and sustained with crystal violet (1% in absolute ethanol). Cell survival was detected by counting the colonies containing > 50 cells. The survival fraction of cells of each irradiation dose group was determined as a plating efficiency of the irradiated cell divided by that of the non-irradiated control.

*Cell Viability and cell proliferation assay*

Stale transfected MDA-MB-468 cells were seeded in 96 wells plates and cultured for 24 h at 3.103 cells /100µL/well. For cells viability analysis, cells were seeded and immediately irradiated with a single dose of IR (0, 2,4,6, and 8 Gy). At 72 h post-incubation, 10 µL of CCK-8 was added to each well and incubated at 37^o^C for 1 h to 2 h following by their optic density (OD) reading at 450nm. For cell proliferation analysis, cells were seeded and irradiated with a single dose of IR (0 Gy and 2Gy). At an appropriated time (0 h, 6 h, 12 h, 24 h, 48 h, and 72 h) post- incubation, 10 µL of CCK-8 was added to each well and incubated at 37^o^C for 1 h to 2 h. The plates were read at 450nm by using a 96 well plate reader. Each experiment was repeated three times. 

*Cell Cycle and Cell apoptosis analysis*

Cell cycle was studied on stable transfected triple-negative breast cancer cells without and with 6Gy irradiation. At 72 h post-culture, the 5.105 cells plated in 6 wells plate were harvested, fixed in 70% ethanol for 30 min and stained with 500 µL of propidium iodure (C1052, Beyotime, China) for 30 min at 37^o^C .

Apoptosis analysis was performed by using FITC-Conjugated annexin V/Propidium iodide method (Annexin V-FITC/PI apoptosis Kit best bio/ China) according to the manufacturer’s instructions. Samples were analyzed by flow cytometry (FACS, AriaIII, and BD, USA) and all tests were repeated three times (in Triplicate). 

*Statistical Analysis*

Statistical analysis was performed with GraphPad Prism 5.0; the comparison of multiple groups was performed with one way ANOVA and Turkey’s method. Data are expressed as mean ± Standard Deviation (SD) and P< 0, 05 was considered as an indicator of statistical significance. 

## Results

*Down-regulation of TMPRSS4 increased the radiosensitivity of MDA-MB-468 cells*

**Figure 1:**
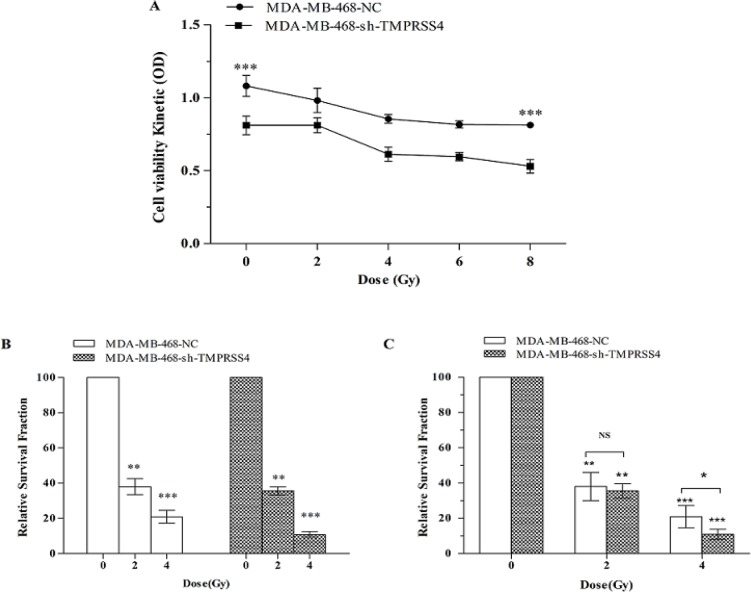
Down-Regulation of* TMPRSS4* Expression Enhances TNBC Radiosensitivity. Stable transfected MDA-MB-468 cells were exposed to different dose of IR (0, 2,4,6,8 Gy) followed by cell viability evaluation using CCK-8 assay (Figure 1A). The stable transfected MDA-MB-468 cells were irradiated at dose 0, 2 and 4Gy following by the counting of colony development (Figure 1B, C). Data represent mean ± Standard Deviation (SD). *P<0.05; **P<0.01 and ***P<0.001

To explore the role of* TMPRSS4* in triple negative breast cancer cells radiosensitivty,* TMPRSS4* was down-regulated by transfecting* TMPRSS4* silenced lentivirus vector in MDA-MB-468 breast cancer cell line which has been reported to overexpress* TMPRSS4* (Cheng et al.,2013; Li et al.,2017; Assani et al., 2019). Colony formation assay and CCK-8 assay was used for performing cell viability analysis. The CCK-8 test indicated that IR decreased the viability of the cell in a dose-dependent manner (Figure 1A) associated with the high decrease at 8 Gy in MDA-MB-468-shTMPRSS4 groups compared to MDA-MB-468-NC [(53.00% ± 4.6% vs 82, 22% ± 2.3%); Figure 1A;***P< 0.001)]. Moreover, colony formation assay showed that* TMPRSS4* silencing significantly decrease colony number (reported by relative survival fraction) in irradiation dose-dependent manner (0,2 and 4Gy) with a high decrease at 4 Gy in MDA-MB-468-shTMPRSS4 groups compared to MDA-MB-468-NC (Figure 1B,***P<0.001; Figure 1C, *P<0,05) This result suggests that* TMPRSS4* silencing improves TNBC radiosensitivity.

*Effect of radiation on cell proliferation in control and TMPRSS4 down-regulated TNBC cells*

**Figure 2:**
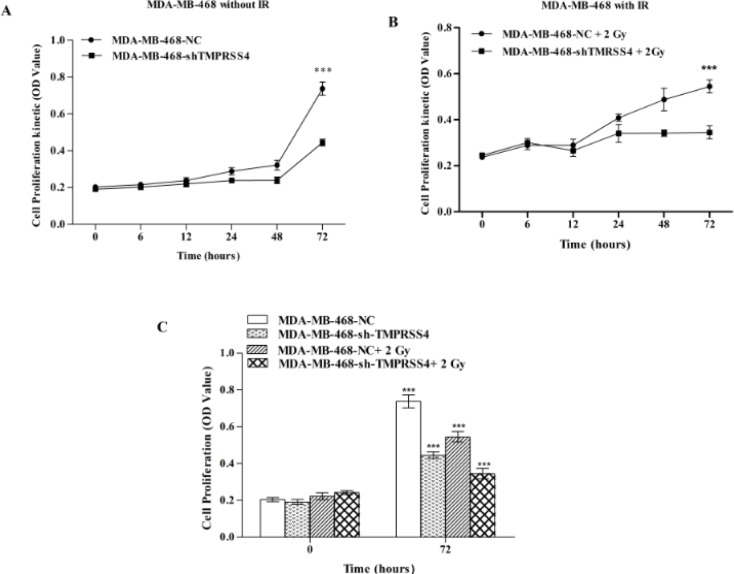
*TMPRSS4* Silencing Enhanced TNBC Cell Proliferation Reduction Mediated by IR. The CCK-8 assay was used to detect reduction of the proliferation capacity of stable transfected MDA-MB-468 cell without IR (Figure 2 A,E) and with 2 Gy IR (Figure 2 B, C). MDA-MB-468 cell showed a remarkably cell proliferation reduction at 72h post-IR in combined treatment groups than shTMPRSS4 or 2 Gy IR alone (Figure 2C). Values are indicated as mean ± SD of three independent experiments

To examine the synergic effect of* TMPRSS4* down-regulation and 2 Gy irradiation on TNBC proliferation, CCK-8 assay was used and MDA-MB-468 proliferation ability was determined in shTMPRSS4, 2 Gy irradiation and shTMPRSS+2 Gy IR treatment groups. Our results showed that* TMPRSS4* inhibition associated with 2 Gy IR significantly reduced the MDA-MB-468 cells proliferation ability with high reduction in MDA-MB-468-shTMPRSS4 cells compared to control [(34,52% ± 2,8 % vs 73,81 % ± 3,5 %); Figure 2C;***P<0.001] than shTMPRSS4 [(44,49 % ± 1,8% vs 73,81 % ± 3,5 %); Figure 2A;***P<0.001] and 2 Gy irradiation alone [(54,52 % ± 2,8 % vs 73,81 % ± 3,5 %); Figure 2B;***P<0.001]. This result suggest that* TMPRSS4* down-regulation enhances cell proliferation reduction induced by 2Gy irradiation Effect of radiation on cell cycle in control and* TMPRSS4* down-regulated TNBC cells. 

**Figure 3:**
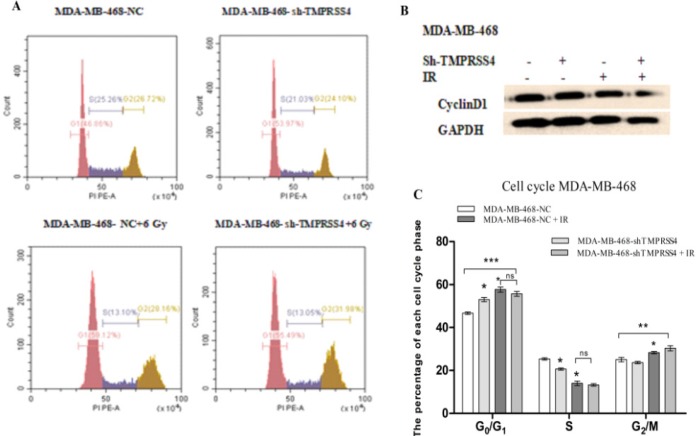
Down-Regulation of* TMPRSS4* Expression Improved MDA-MB-468 Cell Arrested in G2/M Phase Induced by 6 Gy IR. Cell cycle analysis was performed by utilizing Flow cytometer analysis in stable transfected MDB-MB-468 cells without IR (Figure 3A, B) and with 6 Gy IR (Figure C, B). Experiments were performed in triplicate (***P<0.001). Western Blot was also performed regarding the expression of CyclinD1 related to cell cycle pathway (Figure3D).

To assess the effect* TMPRSS4* inhibition in radiation induced cell cycle process impairment, the stable transfected MDA-MB-468 cells and irradiated with 0 and 6 Gy IR were harvested for cell cycle analysis through flow cytometry analysis and western blot analysis of *cyclinD1* expression. Our results showed a significant increase of the rate of the cells arrested in G2/M phase in MDA-MB-468-sh-TMPRSS4 associated with 6 Gy irradiation groups [(30,33% ± 1,15 % vs 19,60 % ± 1,21 %), Figure A,C; ***P<0.001)] than 6 Gy IR treatment alone [ (28,33 % ± 0,57% vs 19,60 % ± 1,21 % Figure 3A,C; ***P<0.001). Moreover, the *cyclin D1 *expression was significantly inhibited in* TMPRSS4* silencing groups than control groups after 6 Gy irradiation (Figure 3B). Our results demonstrated that* TMPRSS4* silencing increased G2/M cell cycle arrested induced by IR which may due to* cyclin D1* inhibition.

*Effect of radiation on cell apoptosis in control and TMPRSS4 down-regulated TNBC cells*

**Figure 4:**
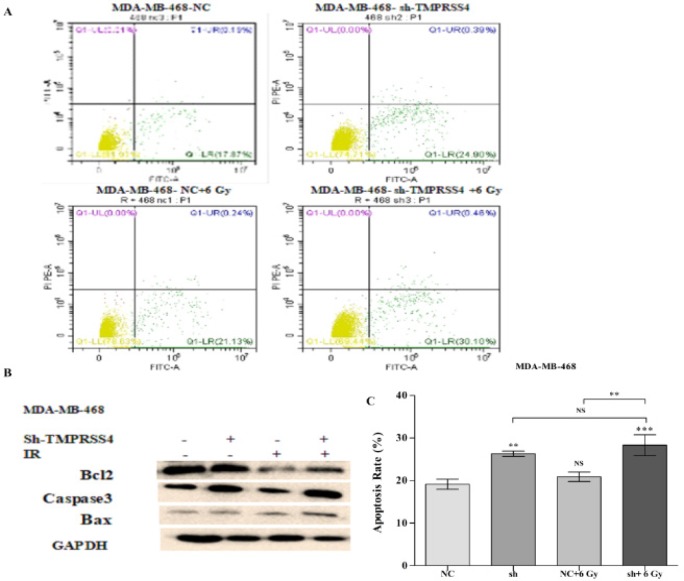
Inhibition of* TMPRSS4* Expression Enhanced IR Induced Cell Apoptosis in MDA-MB-468 Cells. The cell apoptosis was detected via the Annexin V staining assay in stable transfected MDA-MB-468 cells without (Figure 4A, B) and with (Figure 4C, B). Values represent the mean ± SD of three independent experiments (***P<0,001). Western Blot analysis of certain protein (Bax, Bcl2, and Caspase3) related to apoptosis pathways was also investigated in a stably transfected cell under and not 6 Gy IR (Figure 4D)

The impact of inhibition of* TMPRSS4* expression on radiation induced cell apoptosis was studied by performing Annexin V assay and western blot assay for checking the expression of* Bcl2*, *Bax* and *caspase3*. The apoptotic index was significantly increased in MDA-MB-468-shTMPRSS4 groups under irradiation [(28,33 % ± 2,44% vs 19,13 % ± 1,18 %); Figure 4A,B ;***P<0.001] associated with over-expression of pro-apoptotic protein (*Caspase 3*, *Bax*) and down-regulation anti-apoptotic protein (*Bcl2*) (Figure 4C) than in shTMPRSS4 groups [( 26,29 % ± 0,62% vs 19,13 % ± 1,18 %); Figure 4C,B ;**P<0.01]. Few percentage of MDA-MB-468 cells underwent apoptosis in 6 Gy irradiation treatment alone groups compared to control [( 20,89% ± 1,09% vs 19,13 % ± 1,18 %); Figure 4A,B,C ; P (NS)]. This data demonstrate that* TMPRSS4* inhibition improved 6 Gy IR induced MDA-MB-468 cells apoptosis.

**Figure 5:**
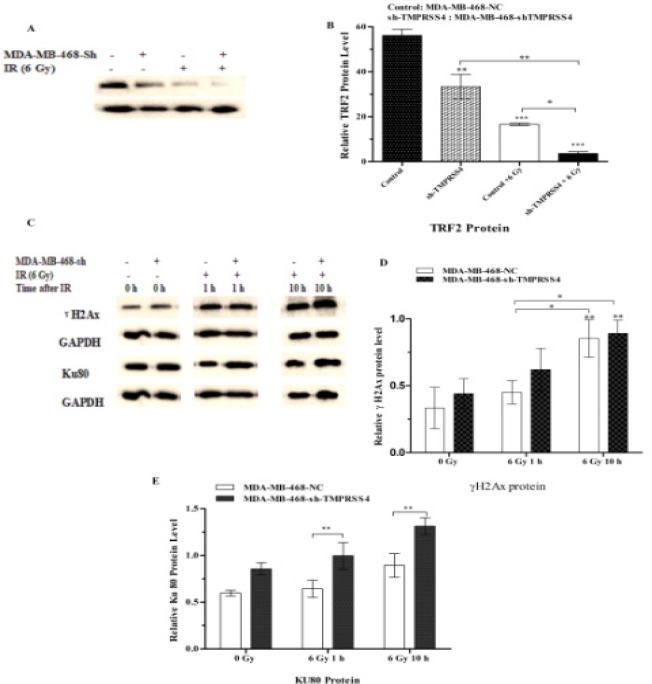
*TMPRSS4* Silencing Improved the DNA Damage Induced by IR, Delays DNA Damage Repair. Western Blot was performed to detect in stable transfected MDA-MB-468 cell the expression level of certain proteins TRF2 and (Ku80, ˠH2Ax) related to DNA damage (Figure 5A,B) and DNA damage repair (Figure 5C,D,E).

Down-regulation of* TMPRSS4* enhances DNA damage and delays DNA damage repair induced by IR in TNBC DNA damage is the most important target of radiation induced cell damage and TRF 2 degradation is reported to illustrated the DNA damage response (Kwon et al., 2013; Zhang et al., 2006). To investigate whether* TMPRSS4* silencing resulted in a change in IR- induced TNBC DNA damage, *TRF2* expression level using western blot was evaluated in MDA-MB-468-shTMPRSS4 and its control with and without 6 Gy IR. TRF2 levels is significantly decreased when radiation was combined with* TMPRSS4* silencing than radiation alone or* TMPRSS4* silencing alone (Figure 5A,B; ;***P<0.001 ). *Ku80* and *ˠH2Ax* expression were investigated at 1 h and 10 h post- IR for DNA damage repair analysis. The expression of *Ku80* and *ˠH2Ax* is continuously increased at 1h post and 10h post- IR with the high increase in MDA-MB-468-shTMPRSS4 groups (Figure 5.A, B, C); suggested that* TMPRSS4* silencing increased the DNA damage induced by IR and delayed the DNA damage repair. Relative gray value = Gtarget protein/GGAPDH (G: gray value) *P<0.05. The experiments were repeated three times independently.

## Discussion

Radiotherapy is one of the most effective breast cancer treatment strategies which significantly improves the therapeutic outcome and survival of breast cancer patients (Moran et al., 2015; Bartelink et al., 2001; Fisher et al., 1995; Cun et al., 2013). Resistance to radiation represents one of the important challenges for breast cancer radiotherapy effectiveness including triple negative breast cancer (Ko et al., 2018; Qi et al.,2017; He et al., 2018) which represent the most radio-resistant breast cancer (He et al., 2018; Kyndi et al., 2008). In the present study, found that* TMPRSS4* inhibition increased TNBC cell radio-sensitivity (Figure 1A, B, C) and improved the cell proliferation reduction mediated by IR (Figure 2C). Moreover, the cell cycle is an important step in cancer development (William et al., 2012; Senderowiez et al., 2002) and cancer treatment such as radiotherapy (Assani et al., 2019; Pawlik et al., 2004). Additionally, *cyclin D1* is one of the proteins related to cell cycle pathway (Casimiro et al., 2012) and radiotherapy (Shimura et al., 2011). Our results indicated that TMPRSS4 silencing increased the cell arrested in G2/M phase mediated by irradiation associated with* cyclinD1* expression inhibition. This suggested that TMPRSS4 silencing enhanced the TNBC cell radio-sensitivity via cell cycle arrested in G2/M phase (Figure 3B,C). It has been reported that *TMPRSS4 *silencing increased cell arrested in the G2/M phase (Assani et al., 2019), ionizing radiation also caused cell arrested in the G2/M phase (Zhao et al., 2019; Dillon et al., 2014; Yan et al., 2007; Luo et al.,2018), *cyclin D1* is induced in the G2 phase under Ras pathways control in rapidly proliferation cell (Sherr et al., 2002), *cyclinD1 *down-regulation sensitize cell to irradiation (Su et al., 2015; Marampon et al., 2016) and cells are more radiosensitive at G2/M phase (Palik et al., 2004). This suggested that TMPRSS4 down-regulation improved TNBC cell radio-sensitivity may be due to *cyclin D1* inhibition and accumulation of G2/M arrested cells. However, further studies are needs to more investigate the implication of the Ras pathway in relationship between *cyclin D1*, *G2/M*, and radio-sensitivity of TNBC cells.

Otherwise, cell apoptosis is one of the keys even in cell life (David et al., 1999) and could be a target of cancer treatment strategy or cancer treatment adjuvant such as radiotherapy (Du et al., 2006; Liu et al., 2011). 

The pro-apoptotic protein (Bax and Caspase3) and the anti-apoptotic protein (*Bcl2*) are the kinds of the proteins related to cell apoptosis pathway and implicated in cancer radiotherapy (Mackey et al., 1998; Dong et al., 2016; Kaliberov et al., 2002). In the present study, we found that *TMPRSS4* down-regulation improved the apoptosis effect of ionizing radiation on triple negative breast cancer (Figure 4A, B) associated with inhibition of expression of *Bcl2* and increased of Bax and Caspase3 (Figure 4C). Our previous study reported that *TMPRSS4 *expression inhibition induced cell apoptosis following by *Bax *and *Bcl2 *expression modulation (Assani et al., 2019) and numerous studies reported that IR promoted cells apoptosis (Assani et al., 2019). Moreover, inhibition of *Bcl2* and up-regulation of *Bax* and *caspase3 *expression were also reported to be associated with increased cancer radiosensitivity through an increase of cancer cell apoptotic index (Jiang et al., 2016). Nevertheless, the low rate of MDA-MB-468 cells underwent to apoptosis in irradiation alone groups may be due to the lack of estrogen receptor (ER) in MDA-MB-468 cells as it has been that ER transfection enhanced radiosensitivity of TNBC cells via additional apoptosis induction (Chen et al., 2017). Based on these data, TMPRSS4 silencing enhanced TNBC radiosensitivity may be due to increase of apoptotic index following by *Bcl2*, *Bax* and *caspase3 *expression impairment The DNA damage is one of the important effects of ionizing radiation on the cancer cells and the inability of cancer cells to repair that DNA damage characterizes the radiotherapy effectiveness (Alizadeh et al., 2015; Mladenov et al., 2013). *TRF2* and (*Ku80*, *ˠH2AX*) are proteins related to DNA damage (Sharma et al., 2012; Wang et al., 2014) and DNA damage repair. We found that TMPRSS4 silencing enhanced the DNA damage induced by through TRF2 inhibition (Figure 5A, B). Subsequently, the complete repair of DNA damage would occur within 30 min to 5 h with the loss of almost DNA damage protein within 7 h- 8 h post IR (Löbrich et al., 2010; Nazarov et al., 2003; Solovjeva et al., 2009; Svetlova et al., 2007). In the present study, we found that TMPRSS4 inhibition delayed DNA damage repair in TNBC cell lines (Figure 5C, D, E) by continuously increasing* Ku80* and *ˠH2AX* expression at 1 h and at 10 h post-IR. TMPRSS4 down-regulation increased TNBC cell radiosensitivity may due to improve of DNA damage and delay of DNA damage repair. However, the mechanism by which TMPRSS4 down-regulation enhanced IR induced DNA damage and delays DNA damage repair remains more investigate. In conclusion, our results indicated that down-regulation of TMPRSS4 improved triple negative breast cancer radiosensitivity via cells arrested in G2/M phase and increase of cells apoptosis ability. This provide the new information for novel combination of therapies synergic using sh-TMPRSS4 with radiation in patient with TNBC. 
